# Repeated *in vivo* inguinal measurements to estimate a single optimal mesh size for inguinal herniorrhaphy

**DOI:** 10.1186/1471-2482-12-19

**Published:** 2012-10-02

**Authors:** Rannie Rabe, Clarence Pio Rey Yacapin, Brian S Buckley, Jose Macario Faylona

**Affiliations:** 1 Division of Hepatobiliary and Pancreatic Surgery, Department of Surgery, University of the Philippines Manila, Philippine General Hospital, Taft Avenue, Manila 1000, Philippines; 2 Department of Surgery, University of the Philippines Manila, Philippine General Hospital, Taft Avenue, Manila 1000, Philippines

**Keywords:** Inguinal hernia, Herniorrhaphy, Mesh, Low and middle income countries

## Abstract

**Background:**

Inguinal hernia is a common condition and its repair (herniorrhaphy) is one of the most commonly performed procedures in general surgery. The Lichtenstein herniorrhaphy technique is a widely used and effective surgery that uses mesh to reinforce the area of weakness. Although a wide range of mesh sizes are available for use in hernia repair, in low-resource health care settings the provision of multiple products may not be supportable and it may be necessary for the provision and use of a single mesh size. This study aimed to determine whether the recommended 7.0 cm x 15.0 cm size is an appropriate single mesh size.

**Methods:**

In order to determine the optimal mesh size according to recommended surgical practices, in vivo measurements of key dimensions of the inguinal floor were taken in patients undergoing herniorrhaphy.

**Results:**

Measurements were taken in 43 patients: 40 men and 3 women, mean age 43 years (SD 13.6); 39 with indirect hernias, 4 with direct. Allowing for recommended mesh overlaps, the optimal mesh size for provision to be appropriate for the majority of patients was determined to be 8.5 cm x 14.0 cm, 21% wider than the mesh size currently recommended for use in Lichtenstein herniorrhaphy.

**Conclusions:**

An appropriate size for routine provision in low-resource settings, or other settings where the provision of several mesh sizes is not supportable, may be 8.5 cm x 14.0 cm.

## Background

Inguinal hernia is a common condition with a lifetime risk of 27% in men and 3% in women, increasing in both sexes with age. Its repair is one of the most commonly performed surgical procedures in general surgery. Rates of inguinal hernia repair have been reported of 10 per 100,000 of the population for the UK and 28 per 100,000 in the USA [1]. Several operative techniques have been developed to treat inguinal hernias. The Lichtenstein tension-free mesh herniorrhaphy is a widely used technique that has been shown to be effective and to have low recurrence rates [2-5].

For each herniorrhaphy case, a surgeon must have on hand a mesh prothesis that will be appropriate. Previous research that has attempted to predict required mesh size pre-operatively according to patients’ body measurements has found no correlation [6]. Thus either a range of products or a single product that can be adapted for each case must be available in the operating room.

It has been recommended that a 7.0 x 15.0 cm mesh prostheses is appropriate for the Lichtenstein technique, and that it should trimmed intra-operatively to a suitable size to cover the inguinal floor and provide overlaps along its points of fixation. Research has suggested that providing adequate overlaps can reduce recurrence rates by compensating for the mesh shrinkage that has been observed in experimental studies [7]. The recommended 15.0 cm mesh length is intended to provide ease of manipulation, with 3.0-4.0 cm being trimmed when it is in place [7]. However, the basis for the 7.0 x 15.0 cm size is uncertain. Currently, there is a lack of published studies of *in vivo* measurements of the inguinal floor where the mesh is laid down.

In adequately resourced settings a supply of different mesh sizes and types can be maintained and the product that is used can be selected intra-operatively. However, in many under-resourced health care contexts worldwide, neither the demand nor the resources exist to support the provision of a range of mesh types and sizes, so that only one mesh size is available to surgeons. In such contexts it is vital for patient outcomes that the size of mesh kept in stock is appropriate for the largest number of patients.

The objective of this study was to determine a single optimal mesh size that is effective clinically, based upon *in vivo* measurements of the inguinal floor where the mesh is laid down and anchored in patients with inguinal hernia.

## Methods

The study was conducted in the Philippine General Hospital, Manila, a tertiary government hospital linked to the national university that largely serves the economically disadvantaged. Some 400 to 500 inguinal herniorrhaphy operations are performed in the hospital in every year [8,9]. The Philippines is a developing country as defined by the International Monetary Fund and has a dual private and public health care system.

Consecutive patients aged 18 years old and over scheduled for elective inguinal herniorrhaphy using the Lichtenstein technique for direct or indirect inguinal hernia at the Department of Surgery of the Philippine General Hospital were invited by a single surgeon to participate. Patients with recurrent inguinal hernia were excluded. Written informed consent was obtained from all participants. Recruitment took place between August and December 2008.

**Figure 1:**
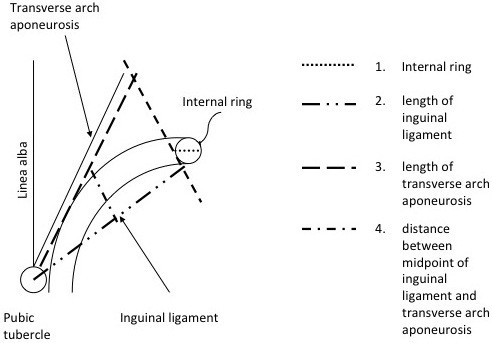
Diagram of the inguinal canal, landmarks and points of measurements.

Intra-operatively, after the opening of the external oblique aponeurosis, the landmark structures were identified and measurements were taken using a sterile ruler. Points of measurement included: (1) diameter of the internal inguinal ring, (2) length of the inguinal ligament from the pubic tubercle up to the inferior border of the internal inguinal ring, (3) length of the transverse arch aponeurosis from the pubic tubercle up to a point at the level of the inferior border of the internal inguinal ring, and (4) the distance between the midpoint of the inguinal ligament and the transverse arch aponeurosis (Figure 1).

Ethical approval for the study was granted by the Ethics Committee of the Expanded Hospital Research Office (EHRO) of the Philippine General Hospital.

## Results

Of forty-five patients invited to participate, forty-three (95.6%) consented to inclusion in the study: 40 males and 3 females with a mean age of 43 years old (SD 13.6). Thirty-nine had indirect inguinal hernia and 4 patients had direct inguinal hernia. Table 1 presents the average measurements of the landmarks around the area where the mesh is laid down.

**Table 1:** Mean measurements of fixation landmarks with standard deviations, ranges and 95% confidence intervals

	**Mean (cm)**	**SD**	**Range (cm)**	**95% CI**
Diameter of internal inguinal ring	2.2	0.7	1.0 – 3.5	2.0, 2.5
Inguinal ligament	5.0	0.7	4.0 – 6.3	4.7, 5.5
Transverse arch aponeurosis	5.4	0.6	4.0 – 7.2	5.2, 5.9
Length between the midpoint of the inguinal ligament and the transverse arch aponeurosis	4.0	1.2	3.0 – 6.5	3.5, 4.5

These measurements suggest that the mean size of the inguinal floor that requires mesh support in the Lichtenstein technique is 4.0 x 5.4 cm. However, studies have shown that it is normal for a mesh to shrink up to 4–7% in 3 months and as much as 20% in 10 months after implant, although the degree of shrinkage can vary between mesh types [10,11]. To allow for shrinkage and reduce recurrence of hernia, it has been recommended that overlaps be allowed on the points of fixation of the mesh of 3.0-4.0 cm beyond the transverse arch aponeurosis, 2.0 cm beyond the pubic tubercle and 5.0-6.0 cm beyond the inferior border of the internal inguinal ring [10]. Adding 4.0 cm to allow for these overlaps to the mean measurement between the midpoint of the inguinal ligament and the transverse arch aponeurosis gives a mesh width of 8.0 cm, or 8.5 cm if the highest value of the confidence interval is used. Adding 8.0 cm to the length of the transverse arch aponeurosis gives a mesh length of 13.4 cm, or 13.9 cm if the highest confidence interval value is used. Thus the measurements recorded in this study suggest a mesh size of 8.5 x 14.0 cm appropriately trimmed to suit each patient’s anatomy, would be adequate for laying down in the vast majority of cases.

## Discussion

Proper mesh size is important in preventing recurrence. Intra-operative observations in recurrent hernia cases have revealed that the mesh slipped away from its medial fixation [12]. This happens more commonly where a mesh is too large or too small: a large mesh can wrinkle, slipping from where it is anchored; shrinkage of a mesh that is too small can result in its being released from its points of fixation due to tension [13].

Manufacturers worldwide produce meshes in a wide range of sizes and of styles. The availability of a range of products may be desirable and may provide some benefit where it is supportable. However, day-to-day budgeting realities in many contexts, especially in public hospitals in developing countries, mean that hospital purchasers must identify an affordable single product or limited range of products for use for all cases.

In terms of mesh length, the size determined by this study is 6% shorter than the 15.0 cm mesh recommended for use in the Lichtenstein technique. However, the 8.5 cm mesh width determined by this study is 21% wider than the recommended 7.0 cm. That the recommended mesh size may be too narrow in a proportion of the patient population is worrying as this has potential clinical implications: where the mesh is too narrow, in these patients it will not be possible to provide the size of overlap that is recommended to prevent recurrence.

These calculations have used the higher of the 95% confidence intervals to offer some degree of certainty that the true mean has been taken into account. Consideration of the full range of observed measurements further highlights that while the recommended length is appropriate, the recommended mesh width is a cause for concern in some cases. Addition of an 8.0 cm overlap allowance to the highest observed transverse arch aponeurosis measurement in this study results in a highest recommended mesh length of 15.2 cm, which is in line with the 15.0 cm recommendation. However, addition of a 4.0 cm overlap allowance to the observed 6.5 cm width suggests that in some cases a mesh as wide as 10.5 cm may be needed, 50% wider than the recommended mesh size.

A prospective trial would be needed to determine what patient benefit, if any, would result from the adoption of 8.5cm x 14cm as the standard flat mesh size. However, it may be unlikely that such a trial will be conducted, since manufacturers are not required to produce such evidence. In addition, since the provision of a single flat mesh size for all patients is most likely to be the norm in low-resource settings, it is unlikely that a publicly funded trial is possible. Nevertheless, our findings may be of interest to manufacturers in determining the standard size of meshes they produce and to those who make surgical supply purchasing decisions.

This study is rare in its use of direct *in vivo* measurements of the inguinal floor to determine an optimal dimension for mesh implants. The study also has limitations that must be acknowledged. It is a small sample, but the confidence intervals generated are relatively narrow, indicating consistency in measurements. The population considered is South East Asian (Filipino nationals of varying ethnic origin) and it is uncertain whether there may be regional variations in anatomical measurements that should be considered. That said, previous research has found no significant correlation between inguinal canal measurements and patients’ weight, height and body mass index [6]. Only a small proportion of patients included had direct inguinal hernias. Further studies in larger samples of different ethnic origin are recommended, as are studies in patients with direct and indirect inguinal hernia in order to determine whether different optimal mesh sizes exist for the two indications.

## Conclusions

It appears that the recommended size of mesh implants for use in inguinal herniorrhaphy may often be too narrow, potentially undermining the surgery’s clinical effectiveness and increasing the risk of recurrence. The implications of this finding are of particular importance in low-resource settings, where the provision of a range of mesh types and sizes may not be possible. The direct *in vivo* measurements reported by this study suggest that the provision of standard flat mesh implants of 8.5 x 14.0 cm would be appropriate for use in the majority of inguinal herniorrhaphy cases.

## Competing interests

None of the authors has any conflict of interest to declare.

## Authors’ contribution

RR conceived the study, collected the data and contributed to writing early drafts and approved the final draft. CPRY collated data, contributed to the interpretation of the data, contributed to writing early drafts and approved the final draft. BSB contributed to the interpretation of the data and wrote advanced drafts and the final draft. JMF conceived and oversaw the research and approved the final draft. All authors read and approved the final manuscript.

## Funding

No funding was received for the study. The authors were supported by their institutions.

## Pre-publication history

The pre-publication history for this paper can be accessed here:

http://www.biomedcentral.com/1471-2482/12/19/prepub
